# IGV-001 cellular immunotherapy for newly diagnosed glioblastoma: overcoming the logistic challenge

**DOI:** 10.3389/fonc.2025.1556450

**Published:** 2025-03-18

**Authors:** Eric T. Wong, Deus Cielo, Konstantina Svokos, Curt Doberstein, Prakash Sampath, John E. Donahue, Michael Punsoni, Nuno Rodrigues, Francesca Rothell, Robert Edwards, Elaina Wang, Tori Riccelli, Carlin Chuck, Elias A. Shaaya, Rahul Sastry, Rohaid Ali, Belinda Shao, Hael Abdulrazeq, Felicia W. Sun, Joshua Feler, Santos E. Santos Fontánez, Natalie Amaral Nieves, Cody Dobertsein, Jennifer Dailey, Christine Yu, Sasmit Sarangi, Heinrich Elinzano, Jerrold L. Boxerman, Esther Yu, Howard Safran, Attila A. Seyhan, Wafik S. El-Deiry, Sharonda Keith, Ziya L. Gokaslan, Clark C. Chen, Athar Malik

**Affiliations:** ^1^ Division of Hematology/Oncology, Department of Medicine and Brown University Health Cancer Institute, Rhode Island Hospital and The Warren Alpert Medical School of Brown University, Providence, RI, United States; ^2^ Department of Neurology, Rhode Island Hospital and The Warren Alpert Medical School of Brown University, Providence, RI, United States; ^3^ Department of Neurosurgery, Rhode Island Hospital and The Warren Alpert Medical School of Brown University, Providence, RI, United States; ^4^ Department of Radiation Oncology, Rhode Island Hospital and The Warren Alpert Medical School of Brown University, Providence, RI, United States; ^5^ Legorreta Cancer Center at Brown University, Providence, RI, United States; ^6^ Division of Neuropathology, Department of Pathology, Rhode Island Hospital and The Warren Alpert Medical School of Brown University, Providence, RI, United States; ^7^ Department of Diagnostic Imaging, Rhode Island Hospital and The Warren Alpert Medical School of Brown University, Providence, RI, United States; ^8^ Department of Pathology and Laboratory Medicine, The Warren Alpert Medical School of Brown University, Providence, RI, United States

**Keywords:** IGV-001, cellular immunotherapy, glioblastoma, logistics, bio-diffusion chambers

## Abstract

**Background:**

IGV-001 is a type of cellular immunotherapy currently being investigated for treating glioblastoma (NCT04485949). It uses the patient’s tumor to elicit an autologous immune response.

**Methods:**

The process involves (i) craniotomy for maximum safe resection of the glioblastoma, (ii) *ex-vivo* treatment of the tumor with an anti-sense oligodeoxynucleotide against insulin-like growth factor 1 receptor followed by irradiation, (iii) placement of the treated tumor in multiple bio-diffusion chambers, which are implanted into the patient’s abdominal sheath to elicit an immune response, and (iv) explantation of the chambers 48 hours later. The clinical trial was open at 32 sites in the United States, and eligible subjects were randomized in a 2:1 ratio to receive bio-diffusion chambers containing either conditioned glioblastoma tissue or a placebo. Patients subsequently proceeded to standard-of-care treatment with concomitant radiation-temozolomide, followed by 6 cycles of adjuvant temozolomide.

**Results:**

The execution of the IGV-001 protocol procedure is complicated and involves a multi-step process requiring mobilization of multiple services within the cancer center of a tertiary care hospital, including neurosurgery, neuro-oncology, radiation oncology, neuroradiology, cancer clinical trial office, and operating room personnel to fulfill the pre-specified protocol requirements in a timely fashion.

**Conclusions:**

We have learned a great deal in the process of developing and executing our internal procedures for this clinical trial. Our description of the IGV-001 protocol workflow may serve as a “blueprint” for future implementation of this type of cellular immunotherapy at other centers. We further discuss some of the lessons we have learned during the trial.

## Highlights

- Execution of IGV-001 consists of 5 distinct phases.
- Intraoperative diagnosis of glioblastoma is required and a minimum of 0.6 grams of tumor is needed for manufacturing.
- Bio-diffusion chambers are implanted on Day 2 and explanted 48-52 hours thereafter.

## Introduction

**Figure 1:**
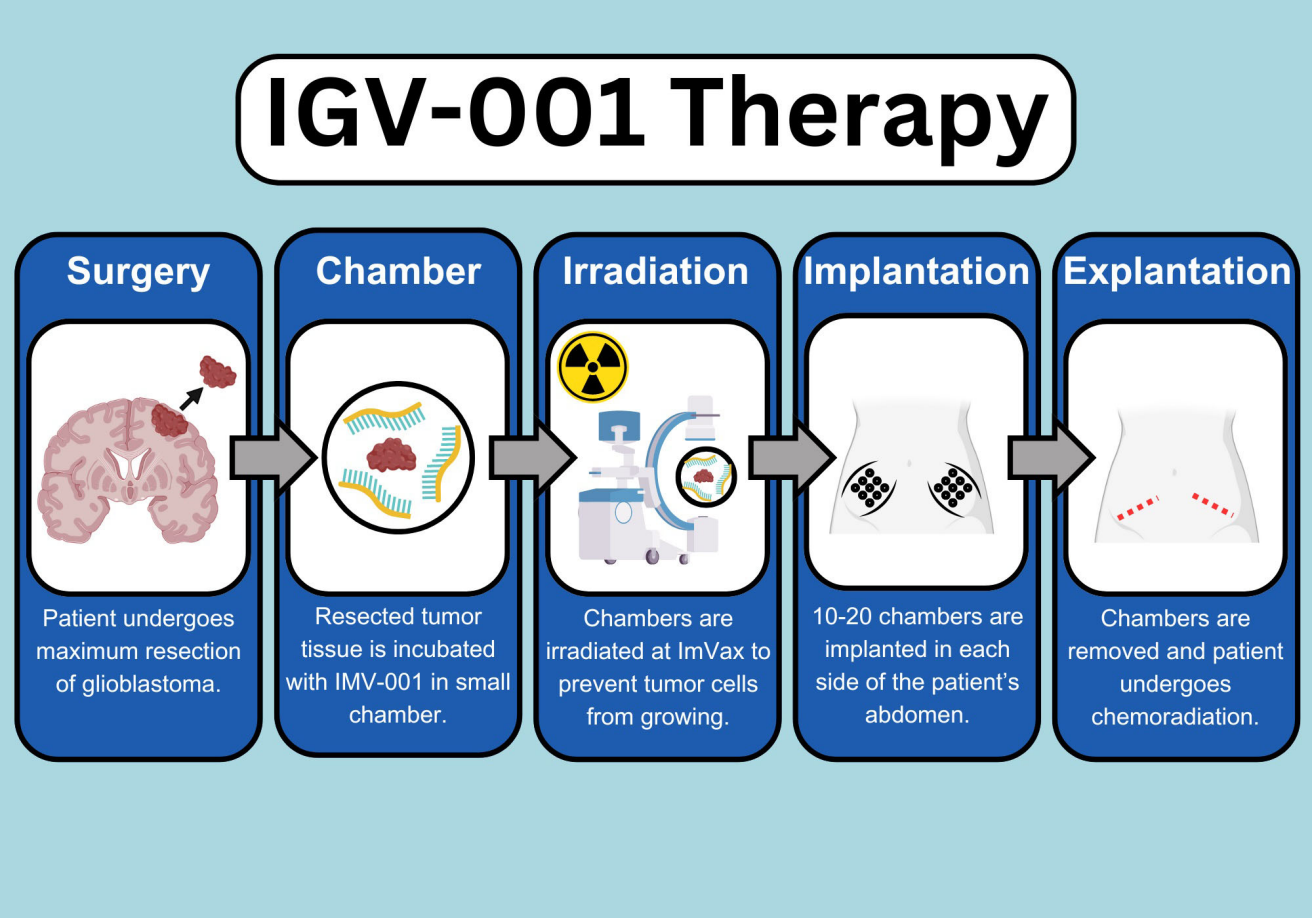
Infographics on IGV-001 execution and protocol schematics. IMV-001, anti-sense oligodeoxynucleotide against IGF-1R. Created using Canva.com and BioRender.com.

Cellular therapies for oncologic and neurologic diseases are ever expanding. This results in an increasing complexity to execute the required procedures for administration of this new type of biologics. In particular, cellular immunotherapy for cancer plays a growing role in controlling diverse types of systemic malignancies. For example, CAR-T requires leukapheresis for the collection of T cells so that they can be engineered to target a specific tumor antigen (1). Likewise, cancer vaccines require tumor harvesting and exposure to immune cells from the adaptive and/or innate immune systems (2). The IGV-001 (**Figure 1**) is a type of cellular immunotherapy that requires the excision of the patient’s primary glioblastoma, followed by *ex-vivo* treatment with an anti-sense oligodeoxynucleotide against the insulin-like growth factor 1 receptor (IGF-1R) followed by irradiation (NCT04485949) (3, 4). The treated tumor cells are then placed in multiple bio-diffusion chambers which are implanted into the patient’s abdominal sheath to elicit an immune response and explanted 48 hours later. This allows for immunologic sensitization and stimulation so that the patient can potentially mount a robust anti-glioblastoma immune response (5).

**Figure 2:**
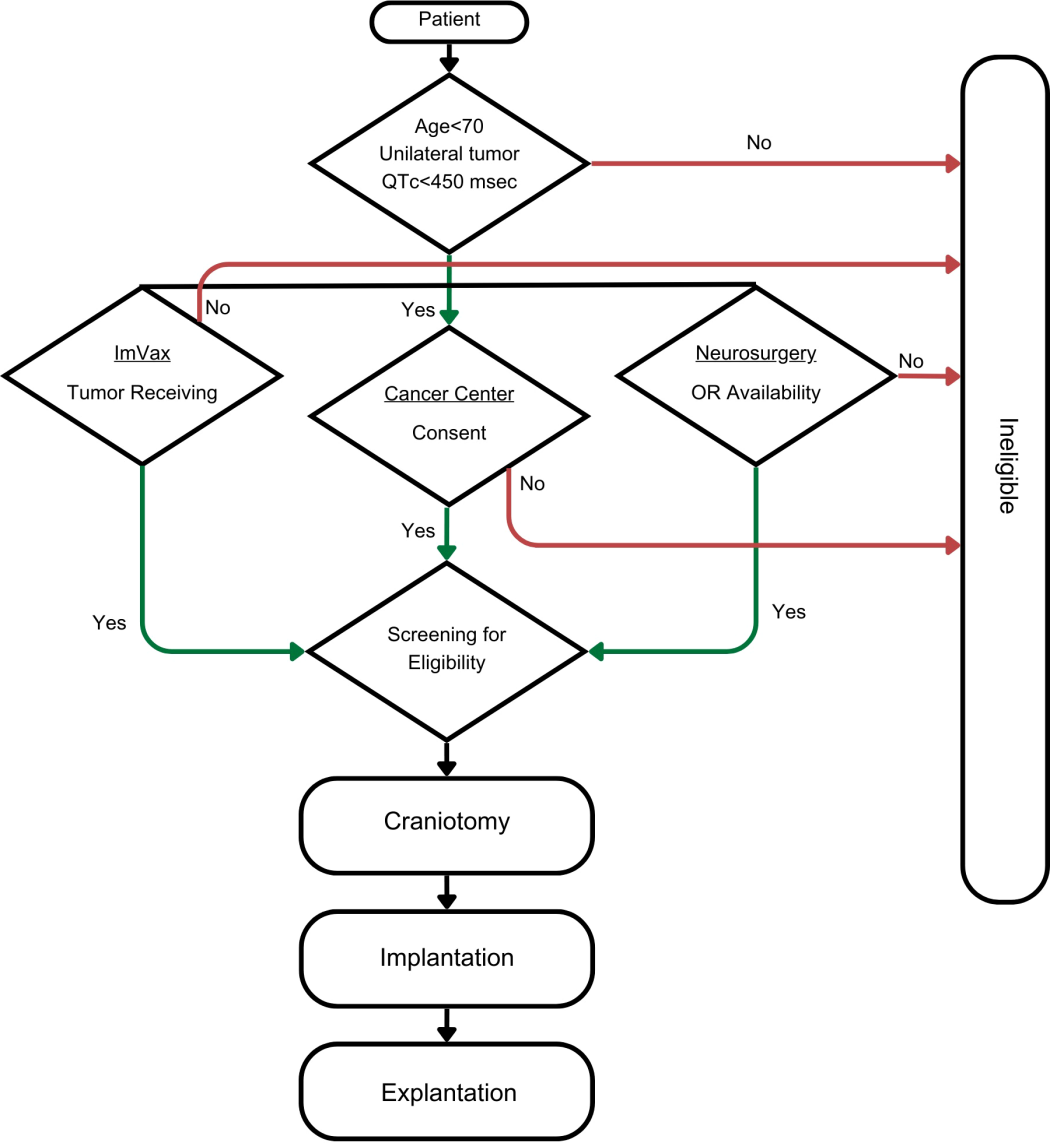
Flow diagram for the execution of IGV-001 procedures.

The execution of the IGV-001 protocol procedure is complex and involves a multi-step process in a tertiary care hospital (**Figure 2**). We implemented the protocol into the following 5 phases: (i) site initiation and protocol approval, (ii) patient identification, (iii) subject registration and screening, (iv) protocol execution, and (v) standard-of-care treatments. Given that the workflow for this clinical trial is different from that of standard-of-care therapy, our description of the IGV-001 protocol workflow at our institution may serve as a “blueprint” for the implementation of this type of cellular immunotherapy in future practice.

## IGV-001, a personalized immunotherapy for glioblastoma

IGV-001 is a personalized, autologous cellular immunotherapy for glioblastoma. Its anti-tumor efficacy was initially demonstrated in mouse models using syngeneic mouse GL-261 (*m*IGV-001), human Y98G and U87 (*h*IGV-001), and patient-derived glioblastoma cell lines (*pd*IGV-001) (6). Subcutaneously implanted bio-diffusion chambers containing irradiated tumor cells treated with antisense oligodeoxynucleotide against IGF-1R generated high mobility group box 1 and extracellular ATP, both of which are immunogenic cell death-associated danger signals that can elicit a potent anti-tumor response (3, 6). Robust interferon-γ induction with concomitant attenuation of immunosuppressive IL-10 and IL-6 cytokines were noted (6). These immunostimulatory changes resulted in the expansion of CD4^+^ and CD8^+^ effector T cells, as well as memory T cells, against *m*IGV-001, *h*IGV-001, and *pd*IGV-001 (6). These encouraging data led to two single-institution studies: (i) a small, phase 1a pilot study (n=12) investigating the feasibility of implantation and explantation of bio-diffusion chambers in humans, and (ii) a phase 1b study (n=33) testing 4 dose levels based on the number of chambers (10 or 20) and duration of implantation (24 or 48 hours) (4, 7). Subjects received the highest dose (20 chambers for 48 hours of implantation) had the longest progression-free and overall survivals, and this dose was used in the randomized, double-blind, placebo-controlled multi-institutional phase 2b study (4, 8). Key inclusion criteria include adults age ≤70, Karnofsky Performance Score ≥70, diagnosis of supratentorial glioblastoma or grade 4 molecular diffuse astrocytoma, bi-dimensional tumor measurement of 4 cm^2^ on magnetic resonance imaging (MRI), at least 2 cc of volume of tumor harvested during surgery, and adequate laboratory parameters and bone marrow reserve (8). Although no specific biomarker was utilized for the pre-selection of participants, age ≤70 and exclusion of specimen with gross necrotic tissue may help identify those likely to respond to this cellular immunotherapy. Accrual was completed in 2024. Rhode Island Hospital is one of the participating institutions and we hereby describe the execution of our workflow in the IGV-001 protocol.

## Site initiation, protocol approval, and activation

After signing the non-disclosure agreement, a copy of the protocol and investigational brochure from the sponsor of the clinical trial (Imvax, Inc.) was sent to the site principal investigator for review. The sponsor then conducted a site initiation visit to determine if our cancer institute had the resources and expertise available to conduct the trial. Once approved, the protocol was scheduled for review by our Scientific Review and Monitoring Committee. In this committee, the merit of the clinical trial concept, the associated protocol procedures, accrual target, and feasibility at the cancer institute were reviewed and debated. Following this step, additional opinions were solicited from research nursing, pharmacy, biostatistics, and the regulatory divisions. At the end of the presentation, a vote was conducted for approval, conditional approval, or disapproval. In the post-approval setting, a site activation procedure was performed in which the site principal investigator, sub-investigators, research pharmacists, research nurses, clinical research assistants, and regulatory division personnel all reviewed the protocol procedures so that the specific roles and responsibilities of each individual were reviewed and identified in the delegation of authority log. Once the protocol was activated, patient identification and enrollment proceeded accordingly.

## Protocol subject identification

Many of our newly diagnosed glioblastoma patients are first seen in the emergency department. Therefore, neurosurgery residents stand at the frontline in the initial evaluation. To educate these residents, a neurosurgery departmental grand round on IGV-001 was held and key points were reinforced in one of the weekly educational sessions, focusing on the specific protocol procedures. This discussion included best practices for glioblastoma resection, pathological assessment during the intraoperative period, quantification of the excised tumor, delivery of the specimen to the sponsor for IGF-1R-specific anti-sense oligodeoxynucleotide treatment and irradiation, implantation of bio-diffusion chambers within the abdominal rectus sheath, and explantation of the chambers. Various phases of clinical trial investigation, including The Food & Drug Administration (FDA) approval process, and post-approval adoption and monitoring of the new treatment were reviewed. The goal being to help residents understand the scientific concepts and rationale behind protocol procedures for IGV-001, as well as the contemporary approval process required by the FDA.

Rhode Island Hospital is the designated level 1 trauma center in the state. This is advantageous because the hospital has an assigned team on call for neurosurgical emergencies and operating rooms are staffed during the weekends and holidays. Protocol execution required precise timing for bio-diffusion chamber implantation on Day 2 after craniotomy and within 24 hours after IGV-001 was manufactured. Similarly, explantation is performed within the window of 48 to 52 hours after implantation. Therefore, when craniotomy occurred on a weekday other than Monday, either the implant or explant would fall on Saturday or Sunday. Because operating room assignment is on a first-come, first-serve basis, the neurosurgical coordinator (S.K.) was notified as soon as a patient agreed to participate in IGV-001. This coordinator subsequently informed the neurosurgery resident to reserve an operating room within the pre-specified timeframe. This ensured neurosurgeon availability and optimal timing for craniotomy, as well as timely execution of the implant and explant bio-diffusion chamber procedures. In addition, the sponsor was notified of potential consents in order to confirm their capability of receiving and processing tumor tissue on the day of craniotomy. Timely manufacturing of the IGV-001 bio-diffusion chambers is necessary as they are to be implanted within the pre-specified timeframe of 48 hours post-craniotomy.

## Subject registration and screening

When a potential subject for the IGV-001 protocol was identified, the research team met with the patient to recapitulate prior discussions made by the neurosurgery service. The neuro-oncologist confirmed tumor resection as the means of diagnosis and advocated for cytoreduction under the condition of maximum safe neurosurgical resection. The standard-of-care option was discussed together with the experimental IGV-001 protocol. The decision to participate was explained to have no effect toward the quality of care that the patient receives, and it was emphasized that protocol participation is entirely voluntary. A copy of the consent form without the signature page was then given to the patient and any family members for review.

After 24-48 hours, the research team approached the patient to address any questions raised and assess the level of interest in participation. If the patient indicated interest, a formal consenting process was performed in a deliberate manner to allow time for questioning and discussion. Once the consent was signed, protocol-specific screening laboratory studies and diagnostic tests were then performed, and the neuro-oncologist investigator reviewed the results for abnormalities. If such abnormalities were present, steps were taken to correct them if possible. The sponsor was notified of a consent once signed to promptly secure a slot in the trial. Finally, a consent note was entered into the patient’s electronic health record with results of the diagnostic studies, fulfilling all eligibility criteria.

## IGV-001 protocol execution

Among the 15 potentially eligible patients pre-screened for IGV-001 (**Table 1**), one had a Karnofsky Performance Score of <70, one had a bi-hemispheric tumor, one underwent prior craniotomy for partial resection of tumor, and 5 declined consent due to uncertainty associated with randomization and the possibility of receiving placebo. Seven individuals signed consent and underwent screening procedures. Four were ineligible due to brain metastasis from lung adenocarcinoma, insufficient (<0.6 grams) glioblastoma tissue, prolonged corrected QT interval (QTc) on the day of craniotomy, and platelet count decrease on subsequent screening labs to below 100 × 10^9^/L secondary to heparin-induced thrombocytopenia. Three subjects successfully underwent craniotomy with sufficient tissue for IGV-001, followed by implant and explant of the bio-diffusion chambers within the pre-specified timeframes per protocol.

**Table 1:** Patient characteristics at Rhode Island Hospital for the IGV-001 protocol.

Patient ID	Screening ID	Status	Age	Gender	Reason for Pre-Screen Failure	Reason for Screen Failure	Date of First Head MRI	Date of Craniotomy	Date of Implantation	Date of Explantation	Start Date of Radiation/TMZ	End Date of Radiation/TMZ
1	N/A	Pre-screen failed	55	Female	KPS <70	N/A	04/12/2023	04/17/2023	N/A	N/A	05/16/2023	06/27/2023
2	003-001	Screen failed	60	Male	N/A	Insufficient tumor	06/07/2023	06/15/2023	N/A	N/A	07/19/2023	08/29/2023
3	N/A	Pre-screen failure		Male	Bi-hemispheric tumor	N/A	N/A	N/A	N/A	N/A	N/A	N/A
4	N/A	Pre-screen failed	57	Male	Prior craniotomy/partial resection	N/A	06/14/2023	06/20/2023 and 07/27/2023	N/A	N/A	08/23/2023	10/13/2023
5	N/A	Pre-screen failed	75	Female	Declined participation	N/A	08/11/2023	08/15/2023	N/A	N/A	09/18/2023	10/06/2023
6	N/A	Pre-screen failed	40	Male	Declined participation	N/A	08/26/2023	08/31/2023	N/A	N/A	10/03/2023	10/04/2023
7	003-002	Screen failed	69	Female	N/A	Lung metastasis	09/10/2023	09/19/2023	N/A	N/A	N/A	N/A
8	N/A	Pre-screen failed	59	Female	Declined participation	N/A	10/03/2023	10/05/2023	N/A	N/A	10/17/2023	12/11/2023
9	003-003	Enrolled	64	Female	N/A	N/A	09/17/2023	09/25/2023	09/27/2023	09/29/2023	11/01/2023	12/15/2023
10	003-004	Screen failed	67	Male	N/A	Prolonged QTc	10/17/2023	10/24/2023	N/A	N/A	12/04/2023	12/22/2023
11	N/A	Pre-screen failed	70	Female	Declined participation	N/A	11/19/2023	11/21/2023	N/A	N/A	N/A	N/A
12	N/A	Pre-screen failed	63	Male	Declined participation	N/A	12/11/2023	12/13/2023	N/A	N/A	01/15/2024	02/23/2024
13	003-005	Screen failed	59	Male	N/A	HIT/low platelet count	12/19/2023	01/11/2024	N/A	N/A	02/07/2024	03/20/2024
14	003-006	Enrolled	67	Female	N/A	N/A	01/10/2024	01/16/2024	01/18/2024	01/20/2024	Patient Declined	Patient Declined
15	003-007	Enrolled	67	Female	N/A	N/A	05/10/2024	05/15/2024	05/17/2024	05/19/2024	06/24/2024	07/02/2024

Three critical characteristics were identified which expedited pre-screening of patients. These characteristics include (i) age ≥18 and ≤70 or outside of this range, (ii) absence or presence of bi-hemispheric disease, and (iii) initial ECG with QTc <450 or ≥450 msec from the emergency department. The latter of each characteristic would preclude patient participation. Rhode Island has an older population and as such, a number of potential patients failed initial screening with an age greater than 70. Per protocol, the tumor must be located solely within one hemisphere without extension across the midline, and this usually involved a review of the first gadolinium-enhanced head MRI. For those tumors located in the frontal, parietal, or occipital lobe, a critical examination of the midline shift is necessary for distinguishing possible mass effect from glioblastoma that shifted the respective cingulate gyrus or parietal white matter across the genu or splenium of the corpus callosum. In our experience, more than half of the ineligible population can be identified through these 3 criteria and subsequently excluded from the trial.

Electrocardiogram (ECG) is a critical procedure during screening, within 24 hours before craniotomy, and prior to explantation. Multiple obstacles related to obtaining these ECGs were noted at our institution. First, the QTc measurements obtained by ECG machines at various parts of the hospital can differ. Therefore, the research team consistently used one machine housed at the clinical trial office, as its proper functioning is checked regularly by our biomedical engineering department. Second, intermittent or continuous electrical noise has been found to interfere with the ECG or rhythm strip (9). Turning off the PureWick™ External Catheter urine collection system, patient motion bed alarm, and/or the telemetry monitor helped to circumvent these particular ECG abnormalities. We found that attention to these details aided the research team in avoiding inadvertent failure of the required ECG protocol procedures.

Five attending neurosurgeons are on-call in weekly rotations for emergencies from brain tumor patients. Once a potential patient was identified for the IGV-001 protocol, the neurosurgeon would discuss pre-operative planning with the patient and the research team. Despite the performance of all neurosurgical procedures under the BrainLab neuronavigation system, pre-operative planning procedure was still important for Patient 2 when his glioblastoma wrapped around the M1 and M2 branches of the left middle cerebral artery within the operculum. This required planning was to ensure the operating neurosurgeon took an optimal approach for maximum safe resection of the tumor. In Patients 10 and 14, the glioblastoma was situated adjacent to the Brocas language area and motor gyrus, respectively. Tumor removal required additional monitoring using intraoperative awake language mapping (**Supplementary Video S1**) and motor evoked potentials for maximum safe resection. All these pre-operative and operative procedures were done to ensure the best possible outcome for the patient while achieving a histological diagnosis and obtaining enough glioblastoma tissue for IGV-001.

All of our subjects’ craniotomies were scheduled as the first case of the day, which started at approximately 08:00 am after they had been transported to the holding suite at 06:00 am for a final pre-operative evaluation by anesthesia and neurosurgery. Our research team ensured timely delivery of the temperature-monitored specimen container and communicated directly with the delivery personnel, typically at 05:30 or 06:00 am. We met each patient in the pre-operative suite to address any questions and ensure collection of the patient’s vital signs and ECG. The research team also entered the operating room and stood by with the specimen container during the craniotomy. The clinical research assistant (CRA) then reviewed the craniotomy procedure and specimen extraction, which was described during site activation, as a refresher with the attending neurosurgeon.

Intraoperative pathological diagnosis of glioblastoma or high-grade glioma is required, based on either tissue smear or frozen section stained with hematoxylin and eosin. The per-protocol specific criteria include the presence of (i) microvascular proliferation, (ii) tumor necrosis, (iii) telomerase reverse transcriptase (*TERT)* promoter mutation, or (iv) epidermal growth factor receptor (EGFR) amplification. The latter two require immunohistochemistry and molecular sequencing, and these procedures cannot be done at the time of resection. However, if the patient had a burr-hole biopsy initially, the status of EGFR amplification and/or *TERT* promoter mutation could be identified prior to resection. In addition, those with glioblastoma arising from a lower-grade astrocytoma, as in diffuse astrocytic glioma, IDH-1 mutant, WHO grade 4, can be diagnosed at the time of resection with (i) microvascular proliferation, (ii) tumor necrosis, or (iii) homozygous deletion of CDKN2A and/or CDKN2B. During the initial review of the tumor specimen, we found that it is helpful for the clinical research team (investigator, research nurse, and CRA) to be present with the per-protocol pathological requirements readily available for review with the on-call neuropathologist. Once a diagnosis of glioblastoma or high-grade glioma is made, the same pathologist can determine how much tissue is needed for the standard-of-care diagnostic workup procedures and then release the rest of the tissue to the research team. A minimum of 1.25 cm^3^ or 2 grams of tumor tissue is required. The CRA then sent the specimen in a container moisturized with saline, properly packed in a temperature-monitored container, and then handed it to the transporter for delivery to the sponsor’s facility for IGV-001 manufacturing.

Implantation of bio-diffusion chambers occurred on Day 2 after craniotomy. This procedure was performed in the operating room also as the first case of the day, which began at 08:00 am. Two sets of manufactured IGV-001 bio-diffusion chambers usually arrive at our institution at 06:00 am in a temperature-controlled container. Each set contained 20 chambers that had been packaged and sealed to guarantee sterility by Imvax in a clam-shell receptacle, which was placed in a transparent plastic bag. Two abdominal incisions were made below the umbilicus followed by separation of the fascia above the abdominal sheath. The clam-shell receptacles were taken out and opened to allow the surgical staff in the sterile field to take the two sets of 20 chambers each for implantation into the two abdominal incisions. The chambers were handled without the use of sharp or pointed instruments. Vicryl ties were attached to each chamber, and these ties were secured together in groups of 5 for implantation into each abdominal incision. The abdominal wounds were then closed in layers, with the skin approximated using running nylon sutures.

The bio-diffusion chambers were explanted within the window of 48 to 52 hours after implant. This procedure was performed in the operating room rather than at the bedside to ensure patient safety and minimize potential adverse events. The explantation was similarly scheduled as the first case of the day due to the timing requirement. Prior to removal, the patient underwent a neurological examination together with pre-specified correlative laboratory studies and ECG. Once timing was established within window, the sutures on the two abdominal incisions were taken out, and the bio-diffusion chambers were removed manually. The space was then irrigated with antibiotic saline followed by closure in layers.

## Standard-of-care treatments and follow-up monitoring

IGV-001 protocol subjects were typically discharged home or to an acute rehabilitation facility after explantation of chambers, on the same day or the day after depending on their neurological and medical status. They subsequently returned to our multidisciplinary brain tumor clinic within 1-2 weeks for a wound check of the scalp and abdomen, as well as evaluation by our radiation oncologists. Radiation and concomitant daily temozolomide at a dose of 75 mg/m^2^/day were started within the pre-specified timeframe of 6 to 8 weeks from the implant date. Therefore, patients had sufficient time to undergo radiation planning and simulation, obtain temozolomide from a specialty pharmacy, receive instruction on chemotherapy intake, and review potential side effects from our pharmacist. Recognizing that tumor control from radiation does not occur until the last 2 weeks of the 6-weeks of radiation, patients are monitored with weekly clinic visits to pre-emptively address potential tumor-related adverse events, including signs of seizures that may require an escalation of anticonvulsant, mobility issues that may necessitate a prolonged dexamethasone taper, and low blood counts from side effects of concurrent medications. The goal was to ensure subjects start radiation and temozolomide on schedule and to continue standard-of-care treatment without adverse events or interruption.

After completion of the initial 6 weeks of treatment, subjects underwent a break of 4 weeks before the start of adjuvant temozolomide. Temozolomide was administered in cycle 1 at a dose of 150 mg/m^2^/day for 5 days in a 28-day cycle, and then escalated to 200 mg/m^2^/day for 5 days monthly in cycles 2 to 6. Patients were monitored with weekly complete blood counts due to a possibility of delayed leukopenia or thrombocytopenia. Pegfilgrastim or romiplostim were the drugs of choice respectively for absolute neutrophil count below 500 cells/dL or platelet count below 20,000/dL. Although these medications are prohibited per protocol, they may be required for patient safety. The rate of decrease in counts was an equally important measure of bone marrow suppression, and the treating neuro-oncologist had to make a clinical judgment call in administering these medications, particularly before the weekend or holidays. Furthermore, the IGV-001 protocol has a pre-specified requirement that each subject must have an absolute neutrophil count of 1,500 cells/dL and a platelet count of 100,000/dL or greater before starting each cycle of adjuvant temozolomide. The overarching objective here is to prevent severe leukopenia or thrombocytopenia that may lead to neutropenic sepsis or bleeding diathesis, respectively.

## Conclusions

Cellular immunotherapy procedures are complicated beyond the standard of care for cancer patients. Therefore, it is important to develop a workflow to ensure adherence to the protocol, so that each procedural component is properly executed. This will require the coordination and cooperation of multiple services within the cancer center of a tertiary care hospital. Our workflow at Rhode Island Hospital is just one example, but it may serve as a “blueprint” for the implementation of IGV-001 or other similar cellular immunotherapies in future practice.

## Data Availability

The original contributions presented in the study are included in the article/**Supplementary Material**. Further inquiries can be directed to the corresponding author.

## Supplementary material

Supplementary Video S1 Craniotomy with awake intraoperative language mapping. https://www.canva.com/design/DAFyroxORyE/kTiGT35vOqPK4Z2jkaS22w/edit.
